# Discrepancy between perceptions and acceptance of clinical decision support Systems: implementation of artificial intelligence for vancomycin dosing

**DOI:** 10.1186/s12911-023-02254-9

**Published:** 2023-08-11

**Authors:** Xinyan Liu, Erin F. Barreto, Yue Dong, Chang Liu, Xiaolan Gao, Mohammad Samie Tootooni, Xuan Song, Kianoush B. Kashani

**Affiliations:** 1 Division of Pulmonary and Critical Care Medicine, Department of Medicine, Mayo Clinic, Rochester, MN 55905 USA; 2 ICU, DongE Hospital Affiliated to Shandong First Medical University, Liaocheng, Shandong 252200 China; 3 Department of Pharmacy, Mayo Clinic, Rochester, MN 55905 USA; 4 Department of Anesthesiology and Perioperative Medicine, Mayo Clinic, Rochester, MN 55905 USA; 5 Department of Critical Care Medicine, Zhongnan Hospital of Wuhan University, Wuhan, Hubei 430071 China; 6 Department of Critical Care Medicine, Division of Life Sciences and Medicine, The First Affiliated Hospital of USTC, University of Science and Technology of China, Hefei, Anhui 230001 China; 7 Health Informatics and Data Science. Health Sciences Campus, Loyola University, Chicago, IL 60611 USA; 8 ICU, Shandong Provincial Hospital Affiliated to Shandong First Medical University, Jinan, Shandong 250098 China; 9 Division of Nephrology and Hypertension, Department of Medicine, Mayo Clinic, 200 First Street SW, Rochester, MN 55905 USA

**Keywords:** Artificial intelligence, Qualitative study, Implementation science, Acute kidney injury, Drug dosing

## Abstract

**Background:**

Artificial intelligence (AI) tools are more effective if accepted by clinicians. We developed an AI-based clinical decision support system (CDSS) to facilitate vancomycin dosing. This qualitative study assesses clinicians' perceptions regarding CDSS implementation.

**Methods:**

Thirteen semi-structured interviews were conducted with critical care pharmacists, at Mayo Clinic (Rochester, MN), from March through April 2020. Eight clinical cases were discussed with each pharmacist (*N* = 104). Following initial responses, we revealed the CDSS recommendations to assess participants' reactions and feedback. Interviews were audio-recorded, transcribed, and summarized.

**Results:**

The participants reported considerable time and effort invested daily in individualizing vancomycin therapy for hospitalized patients. Most pharmacists agreed that such a CDSS could favorably affect (*N* = 8, 62%) or enhance (9, 69%) their ability to make vancomycin dosing decisions. In case-based evaluations, pharmacists' empiric doses differed from the CDSS recommendation in most cases (88/104, 85%). Following revealing the CDSS recommendations, we noted 78% (69/88) discrepant doses. In discrepant cases, pharmacists indicated they would not alter their recommendations. The reasons for declining the CDSS recommendation were general distrust of CDSS, lack of dynamic evaluation and in-depth analysis, inability to integrate all clinical data, and lack of a risk index.

**Conclusion:**

While pharmacists acknowledged enthusiasm about the advantages of AI-based models to improve drug dosing, they were reluctant to integrate the tool into clinical practice. Additional research is necessary to determine the optimal approach to implementing CDSS at the point of care acceptable to clinicians and effective at improving patient outcomes.

**Supplementary Information:**

The online version contains supplementary material available at 10.1186/s12911-023-02254-9.

## Contributions to the literature

- The authors have implemented several educational modules, including quality improvement (PMID 24988421)
- We also developed and implemented acute kidney injury electronic surveillance and prediction (PMID 26070247; 31054606)

## Background

Artificial intelligence (AI) is the simulation of human intelligence in machines that are programmed to think like humans and mimic their actions [1]. In simple terms, AI is defined as “machines that mimic cognitive functions similar to the human mind, such as learning and problem solving” [2]. It has many potential applications in the medical field, including predicting and diagnosing diseases, clinical decision support, medical education, and drug discovery [3–6]. Investigations regarding the AI's role in individualizing drug dosing are limited, but it could enrich the practice with its higher precision and timeliness. Clinical decision support systems (CDSS) hold the promise of helping clinicians make better and more personalized treatment decisions, streamlining workflow, improving outcomes, and reducing healthcare expenditures [7–10].

Vancomycin is widely used to treat serious Gram-positive infections, including methicillin-resistant Staphylococcus aureus (MRSA). The vancomycin therapeutic window is narrow [11, 12]. Overdosing could lead to vancomycin-associated acute kidney injury, and underdosing could lead to antibiotic ineffectiveness and resistance emergence [13–15]. Especially in ICU patients, the complex and dynamic pathophysiology leads to significant inter- and intraindividual variability in vancomycin pharmacokinetics [16, 17]. Studies have found that nearly 2/3 of patients in ICU do not meet the initial target blood concentration of vancomycin [18]. Therefore, there is a need to evolve from a one-size-fits-all dosing approach to tailored regimens designed to improve antimicrobial efficacy and safety [19]. With the advances in AI, attempts have been made to develop dose guidance or plasma concentration prediction models for vancomycin both in and outside the ICU [20, 21]. While these efforts are necessary, AI is only effective if it can be implemented into the workflow at the point of care. Clinical outcomes benefits and technical integration considerations need to be evaluated parallel with the end-user experience. In any clinical scenario, if a very high performing AI-based CDSS is implemented to inform drug dosing, but clinicians are unwilling or unable to adjust drug dosing according to AI recommendations, there could be a potential risk of harm, waste, or confusion.

In the case of vancomycin, we developed an AI-based CDSS tool that uses a comprehensive analysis of patient-specific data to suggest an appropriate regimen for vancomycin (dose and interval) to optimize drug-level target achievement. In this study, we qualitatively assessed pharmacist perceptions and attitudes toward implementing such a CDSS tool using case-based scenarios to evaluate the feasibility and acceptability of implementation.

## Methods

### Setting and participants

The study was conducted at Mayo Clinic in Rochester, MN, from March through April 2020 and reported according to the Standards for Reporting Qualitative Research [22]. This study was deemed exempt by the institutional review board of Mayo Clinic, Rochester, MN (#19–010472). Mayo Clinic is a large academic medical center with 215 intensive care unit beds. Published institutionally endorsed vancomycin dosing recommendations adapted from national guidelines [23, 24] were in place throughout the study. These broadly included weight-based vancomycin doses and intervals informed by the estimated creatinine clearance (eCLcr) based on the Cockcroft-Gault equation. In addition, the care team tailored therapeutic plans according to other patient-specific factors (i.e., the severity of illness, urine output)- consistent with routine clinical practice. The institutional policy provides broad authority for pharmacists to independently select and monitor vancomycin regimens in hospitalized patients. Clinical pharmacists are available seven days weekly from 07:00 to 22:30 in the ICU nursing units and overnight by consultation.

We selected clinical pharmacists involved with hospitalized patients treated with vancomycin via purposive sampling. This study focused on critically ill patients, given their high variability in vancomycin pharmacokinetics and the robust availability of granular patient data in ICUs. Individuals were recruited from different specialty areas that provide care to critically ill patients, including decentralized clinical pharmacists in the ICU and those consulted overnight. Participants were also sampled to attain diversity in levels of expertise and training, from relatively new to the practice to advanced training in individualizing pharmacotherapy.

### Data collection

Interviews were conducted for two months. Two weeks before the interview, a study team member (XL) emailed each eligible pharmacist a recruitment letter describing the reasons for doing the study. Those who provided informed consent to participate were scheduled for a 60-min semi-structured interview, in person, over the phone, or virtually. No one else was present besides the participants and researchers, and no compensation was provided for participation.

Semi-structured interviews were informed by an a priori developed interview guide created for the study (Supplementary Appendix 1). The interview script was designed to assess the pharmacists' demographics, attitudes toward CDSS implementation and acceptance of its recommendations, and reasons for reluctance when presented with clinical cases. There were three primary sections of the interview script, 1) capturing participants' demographics (age, gender), specialty, experience level, and current approach to dosing and monitoring vancomycin, 2) focusing on participants' impressions regarding implementing a CDSS tool to inform vancomycin dosing, and 3) involvement in eight cases inspired by the real-world experience of patients treated with vancomycin. Pharmacists were asked to recommend a dosing scheme before revealing the CDSS recommendations. We then asked about their willingness to accept the CDSS-recommended dosing regimen. The interview concluded with a discussion about factors that would enhance the acceptability of CDSS recommendations in practice. Interviews were audio-recorded with the permission of each participant, transcribed verbatim, and de-identified. The transcripts of the interviews were then subjected to content analysis for themes. Finally, the summary of each interview was returned to the participant for correction.

### Study aims

The primary objective was to identify factors influencing agreement with and adherence to the CDSS recommendations for vancomycin. Secondary objectives included assessing critical care pharmacists' impressions towards CDSS, interactions with CDSS, acceptance of its recommendations, and reasons for not following the dosing regimen recommended by CDSS.

### Data analysis

Interview transcripts were analyzed using the thematic analysis method described by Braun and Clarke [25]. The following steps were performed in the analysis, 1) transcripts were repeatedly read to ensure familiarisation with the data, 2) XL and XS produced initial codes, 3) emerging themes and subthemes were subsequently generated based on significant patterns in the codes, 4) themes and subthemes were continuously reviewed and refined using the constant comparison approach before being included in the final write-up, and 5) the final themes and subthemes were discussed with all members of the research team to ensure that a consensus was reached.

## Results

### Participant demographics

We recruited 15 pharmacists, 2 of whom dropped out due to loss to follow-up, and finally, 13 of whom completed the interviews (Table 1). The median interview time was 42 min (34 to 52 min). Participants were 31% female, with an average age of 35 (ranging from 27 to 50) years. The majority of participants were critical care pharmacists (*N* = 8). In addition, five interviewees were overnight pharmacists responsible for consultation about critically ill patients. Their levels of work experience, training, and CDSS understanding varied.

**Table 1:** Demographic characteristics

Characteristics (*N* = 13)	n (%)
Gender	
Female	4 (31)
Male	9 (69)
Age in years	
25–30	3 (23)
31–35	6 (45)
36–40	2 (16)
> 40	2 (16)
Pharmacist specialty	
Central Pharmacist	5 (38)
Critical Care Pharmacist	8 (62)
Years of practice	
0–5	5 (38)
6–10	5 (38)
11–15	1 (8)
> 15	2 (16)

### Vancomycin dosing work burden

For patients newly started on vancomycin, most pharmacists (*N* = 10, 77%) admitted the need for 0–5 time reviews of the vancomycin regimen, with 23% (*N* = 3) of pharmacists indicating the need for 6–10 time reviews of the vancomycin regimen. Eight (62%) pharmacists indicated they would need up to 10 min when determining the vancomycin dose for a new patient. Five (38%) pharmacists mentioned needing 11 to 20 min to complete the vancomycin dosing regimen (Table 2).

**Table 2:** Vancomycin dosage methods (*N* = 13)

Question	n (%)
Q5. Assuming you have an average census for patients newly started on vancomycin during your shift, on how many occasions do you review or re-review their vancomycin regimens (across all of these new starts)?	
0–5 times	10 (77)
6–10 times	3 (23)
Q6. Assuming you have an average census for patients stabilized on vancomycin prior to your shift, on how many occasions do you review or re-review their vancomycin regimens (across all of these treated patients)?	
0–5 times	13 (100)
Q7. How long does it take you to determine a dose of vancomycin for a new patient?	
0–10 min	8 (62)
11–20 min	5 (38)
Q8. How long does it take you to review and, if appropriate, revise a regimen for the patients who are already on vancomycin therapy?	
0–5 min	2 (16)
5–10 min	10 (76)
11–15 min	1 (8)
Q9. What percentage of the time do you need to change the dose entered by the ordering provider?	
< 70%	2 (15)
70%-80%	8 (62)
81%-90%	2 (15)
> 90%	1 (8)
Q10. Which formula do you use to dose vancomycin?	
CrCI, by Cockroft-Gault	9 (69)
eGFR_Cr_, by MDRD or CKD-EPI	1 (8)
eGFR_Cr-CysC_, by CKD-EPI	2 (15)
Other	1 (8)

*CrCI* Creatinine Clearance, *eGFR* Estimated glomerular filtration rate, *MDRD* Modification of Diet in Renal Disease, *CKD-EPI* Chronic Kidney Disease Epidemiology Collaboration

For patients on stable maintenance vancomycin doses, all pharmacists indicated that they require 0–5 time reviews of the vancomycin regimen per patient, with 62% (8/13) needing 5 to 10 min per review, and one (8%) needing 11 to 15 min. After reviewing the dosing regimen, 62% (8/13) of pharmacists mentioned changing the vancomycin dosing regimen in 70–80% of patients. To determine the appropriate dosing regimen, 69% (*N* = 9) of pharmacists primarily used the Cockcroft-Gault formula (Table 2).

### Attitudes toward vancomycin CDSS in general

**Fig. 1:**
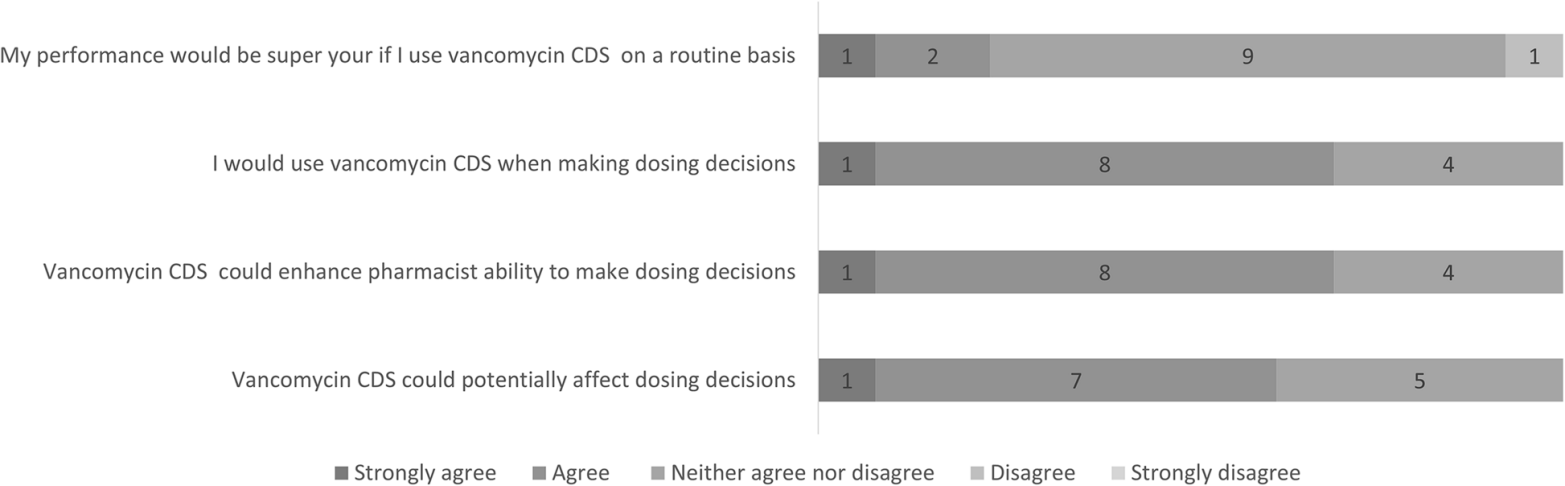
Participants attitudes toward vancomycin dosing clinical decision support system (CDSS)

Most pharmacists (*N* = 8, 62%) agreed or strongly agreed that an AI-based CDSS tool could affect dosing decisions for vancomycin. In addition, most (*N* = 9, 69%) agreed or strongly agreed that CDSS could enhance their ability to make vancomycin dosing decisions, and they would use it in their work. However, 69% (*N* = 9) were neutral on whether their performance would be superior if CDSS was routinely used (Fig. 1).

### Acceptability of vancomycin CDSS

Each of the 13 pharmacists provided a dosing scheme for eight ICU cases who received vancomycin therapy. The total number of cases discussed with all pharmacists was 104. In 88 (85%) cases, the pharmacists suggested a different dosing regimen from the CDSS recommendation (40 cases with lower dosage suggestions and 48 cases with higher dosage suggestions). However, when the CDSS recommendations were revealed, most pharmacist decisions (69/88, 78%) did not adjust the dosing regimen. This was despite generally a positive attitude toward CDSS with comments like "a great project, and it would be really helpful and useful." Others endorsed how CDSS could prompt them to double-check their decision, which could help them make more informed and thoughtful decisions. Still, few were enthusiastic about modifying their previously recommended doses. The provided rationales for their decisions included a distrust and lack of understanding of the justification for the CDSS recommendations, concerns related to the lack of dynamic evaluation or in-depth analysis, incomplete integration of all clinical data, and lack of availability of risk index along with the recommendation (Tables 3 and 4). In the few discrepant cases where the pharmacist was willing to change their doses (19 out of 88, 22%), the primary motivation was the proximity of their dose selection with the CDSS recommendation.

**Table 3:** Pharmacists’ perceptions of AI recommendations

Perception	Theme	Example Quote
Positive	Auxiliary tool	*“If my dose is similar to the AI dose, I probably change it. But again, I'd probably end up splitting the difference rather than just blindly accepting what the models are telling me, I probably would use it as a facet of what we're trying to the dosing tools.”*
	Double-check dosing regimen	“*You know, if I was very different from the AI, I would double-check myself. I would take it out and take it seriously, and maybe I missed something. So I think it would be beneficial even though I may not agree with its dose.*”
	Develop optimal dosing scheme	*“Then, if it's discrepant, I think it would cause me to put some more thought into the patient, so it would help challenge my initial decision, which would help me make a more informed and thoughtful decision.”*
		*“I think I think it's a great project, and I think it would be really helpful and useful because everyone's scope is fairly narrow.”*
	Decision making on uncertain cases	*“I would be on edge and conflicted for this case. With his age, I’m less sure that I would use q8h dosing. But I would take pause to think about it, and AI would help me make the decision”*
		*“I think if, in this case, it's the AI suggested that there was a 70% probability that I was overdosing, I would potentially modify the dose.”*
	Career development	*“AI is a new system. So if we could utilize it, that would also be like another piece to the puzzle that we can potentially use. So yeah, if I knew that the AI was staying me to go higher, I would probably use it.”*
Negative	Self-confidence	*“I was pretty confident that I was right. No amount of computer would be able to say to kind of convince me otherwise.”*
		*“I would say I wouldn't worry that the models indicate me inappropriate, but I like my dosing better.”*
	Mistrust of AI	*“I think if in this case, it's the AI suggested that there was a 70% probability that I was overdosing; otherwise, I won't change my dosage. In short, I don't fully trust it”*
	Unclear rationale for recommendations	*“The reason I wouldn't change my dose scheme is that I don't know why, what factors AI is based on to tell me I'm underdosing or overdosing.”*
		*“I would say no unless I could be given a reason why the AI model says that it's overdosing. I would want to have those responses to what the reasons would be”*

*AI* Artificial Intelligence, *q8h* Every 8 h

**Table 4:** Pharmacists’ willingness to accept AI recommendations

Perception	Theme	Example Quote
**Agree**	Similar dose scheme	*“I could agree with the model recommendation. There really there so close, I don't, although the interval for the model was slightly higher, which, which, you know, sometimes that might be the case.”*
		*“if it more closely resembled my dosing, if it was just a little bit off, I would be more likely to use it.”*
	Concessions to AI	*“If a model told me that dose would be acceptable, I probably would kind of split the difference.”*
	AI is better	*“I would change the dose because I was on the higher side and would want to minimize renal injury. It’s slightly lower than my dose. I prefer the AI’s recommendation and would have chosen that”*
**Disagree**	Don't trust the AI	*“Oh, I think I would follow my dose. I don't think that trained model recommendation because I've given his age and his body weight and is severity illness, I would want to be a little more aggressive.”*
	Lack of dynamic evaluation	*“I do not want to change. I always start patients aggressively and then monitor closely. So even if I start q8h, and then I find that creatinine is worse, or hemodynamics look poor, and they had decreased urine output, I would make a decision to check a level sooner. However, in the ICU, you know, initial creatinine is just a starting point; we have to follow up and double-check and triple-check.”*
	Lack of in-depth analysis	*“I wouldn't want to change my dose at this point until we got cultures back for the simple fact if we don't know what and sensitivities are going to be. She is a very complicated clinical course, and I do not want to make it worse by underdosing in the beginning.”*
		*“This seems to be painful to me, and I would want to have the higher dose. because it's an infection of the knee joint, which means you need a high target range to get a high enough concentration of the drug at the actual site of the infection.”*
	Not all data can be consolidated	*“I wouldn't change it. I will keep it the same. You really need to correlate the numbers you see on the labs with the patient's clinical picture.”*
	Risk index	*“So as a result, I think I won't change my dose unless you told me that I had a 95% chance of overdosing. I'd want to know what that's based on.”*
	Vial sizes	*“Sometimes we think about vial sizes also, so we don't waste medication and ease of dosing.”*

*AI* Artificial Intelligence, *ICU* Intensive care unit, *q8h* Every 8 h

In the final interview question, participants discussed potential methods to enhance compliance with the CDSS dosage recommendations for each patient (Table 5). These included integrating CDSS into electronic health records (EHR), transparency regarding the recommended doses' rationale, including a holistic view of the patient's clinical state, and risk index.

**Table 5:** Methods to enhance pharmacist compliance with AI recommendations

Improvement	Example Quote
Integrate AI into EHR	*“I suggest that this model be integrated into Epic, and please tell me where I could access it from within an order, this will encourage me to use it……, could incorporate the data from the EHR smoothly.”*
	*“I guess, round of the computer system, or at the very least within the Epic, have a quick way to get into this. Myself and a lot of my colleagues don't like to have a lot of buttons to quaking to see the information, so anything that we can do on a quick basis would help us be more compliant with it.”*
Provide a rationale for recommended dose	*“Provide the recommended dose and give reasons why AI recommended this dose. I think this process can make AI more efficient.”*
	*“Because I want to know are we missing something like that, that the system is maybe catching and that we could then use that in future dosing.”*
Show pharmacokinetic values	*“So expected half-life for the patient, what the peak value would be, what the trough value would be, what area under the curve would be within AI’s scheme compared to our scheme. And think that those would both be helpful tools as well to give us some sort of objective sense that my dosing really not appropriate here.”*
Consider trends of the data, not a snapshot in time	*“I want to know that it's taken into account what's happened in the past, that it can kind of foresee what potentially can happen in the future based on Experience or previous cases that can resemble it.”*
	*“Yeah, we're starting antibiotics at the right time, and then all of a sudden they become hypotensive, and the renal hypoperfusion might not be seen for another day or two, but sometimes we have to take that into account so that we don't overdose.”*
Synthesize lab data and provide an overview of patients' clinical state	*“If AI could tell me quickly, you know, the patient's urine output, blood pressure, and their trends, all the clinical things we use to correlate the lab data to what's actually going on real-time……So if the AI could gather that information and save me a lot of clicks.”*
Provide a risk index	*“Good suggestions in this model can give us recommendations and a risk index. If your dose is different from the AI, the risk index can indicate the degree of risk that your dose is overdosing or underdosing.”*
	*“Oh, I think if there was a method by which when we go to enter the dose, there is and the alarm or a warning sign that suggests that the probability that the dose is going to be off is considerable.”*

*AI* Artificial intelligence, *EHR* Electronic health records

## Discussion

This qualitative study is the first in-depth assessment of pharmacists' attitudes toward using an AI-based CDSS tool to enhance vancomycin dosing for hospitalized and critically ill patients. While the clinical providers expressed significant interest in the models, most did not accept changing their dose calculation results when their dose calculation differed from the model's recommended dose.

Despite vancomycin's vital role in treating MRSA infections, a consensus has not been reached on the optimal dosing regimens and pharmacokinetic/pharmacodynamic goals in critically ill patients [26]. Given those critically ill patients in ICUs have varying levels of organ function, therapeutic drug monitoring should be considered to achieve pharmacokinetic goals [27, 28]. Pharmacists spend considerable time reviewing and changing the dosing scheme of vancomycin for patients daily [29]. Our study showed that different pharmacists use different formulae to prescribe vancomycin, resulting in variability in dosing schemes, even though they all consider age, weight, GFR, medical history, and fluid status in their calculations. Additionally, Flannery et al. conducted a recent survey of vancomycin dosing practices among critical care pharmacists [30]. The authors revealed that while pharmacists largely adhere to the 2009 vancomycin dosing guidelines by the American Society of Health-System Pharmacists, the Infectious Diseases Society of America, and the Society of Infectious Diseases Pharmacists [23, 31], they often deviate from these guidelines regarding loading doses, dosing based on patients' weight, and systematically monitoring patients for nephrotoxicity. Developing a vancomycin dosing regimen for critically ill ICU patients is a complicated task involving several variables and often pharmacist judgment calls.

AI is rapidly advancing the field of medicine, which has become a subject of great interest and intense debate. Significant developments based on real-time prediction of adverse events (patient deterioration, acute kidney injury, etc.), personalized drug dosing (antibiotics, etc.), and virtual scribes to help manage patient notes are being implemented [32]. These technologies promise to improve decision-making processes, create more personalized approaches, boost workflow, and reduce healthcare expenditures [7–9, 33, 34]. In the ICU, applications of AI include a collection of data analytics and modeling techniques aimed at generating knowledge from data [6]. We developed an AI-based CDSS tool to address the inconsistent dosing of vancomycin regimen and decrease the incidence of vancomycin-associated nephrotoxicity while maintaining its clinical efficacy.^1^

There are several technological challenges to overcome in building these complex models and turning them into bedside tools, including but not limited to preparing curated real-time databases, handling interoperability issues, and managing incomplete and spurious data artifacts. Medicine is a high-risk environment where deploying inaccurate or poorly calibrated algorithms would be unacceptable [35]. The successful deployment of AI requires the trust and buy-in of all stakeholders [33]. Clinicians are primarily interested in scrutinizing the accuracy of the recommended dosing regimen, the rationale, and the specificity of the recommendation for each patient vs. recommendations given for the entire population [36]. Additionally, as noted by our study participants, the user interface will play a role in integrating CDSS into their workflow. In CDSS development, understanding the attitudes and perceptions of those who will be the primary users is critical. Understanding their hesitancies in incorporating AI into their workflow will inform future iterations of AI for higher clinical compliance and clinician acceptance.

Our interviews with pharmacists who provided vancomycin dosing regimens for patients showed positive attitudes toward the AI as a second opinion and a resource that offers reasons for double-checking their decisions. Still, many pharmacists held distrust, leading to low compliance with the recommendations. The primary reason for low compliance was the black-box nature of CDSS recommendations. In addition, the complexity of some AI algorithms, their lack of transparency, and a widespread lack of prospective validation may lead to lower trust and enhanced concerns [3]. Our study provides valuable insights into improvements that would potentially increase the compliance of pharmacists with the vancomycin-dosing CDSS recommendations. In addition, our findings could be extrapolated to other areas, such as renally eliminated and nephrotoxic drugs.

This study had several limitations. First, our results may not be transferrable considering the small number of participating pharmacists from a single large academic medical center. Second, some interview questions could lead the clinicians and result in biased responses. Third, while the clinicians were informed about the very good overall performance of the AI tool, detailed information regarding the model's features was not shared with them. This notion could have impacted their trust in the model's output, particularly if they disagreed with the model’s recommended dosage.

## Conclusion

While pharmacists acknowledge the advantages of AI, they prefer AI as a supportive tool rather than a decision-maker. AI may have a role in improving their workflow and instilling more support into their practice, but a lack of trust in the AI recommendations could potentially hinder improving compliance. Therefore, to enhance clinicians' compliance with the CDSS tools regarding drug dosing, we suggest the integration of AI into electronic health records and workflow and to improve its transparency regarding its features and performance accuracy. Change management strategies to transform culture would be the next steps toward higher compliance with these digital health solutions in critical care settings.

### Supplementary Information

**Additional file 1: Appendix 1.**

## Data Availability

The script and supplementary materials provide a copy of the verbal consent script and summarize interview data.

## Abbreviations

AI: Artificial intelligence
CDSS: Clinical decision support system
CKD-EPI: Chronic Kidney Disease Epidemiology Collaboration
CrCI: Creatinine Clearance
eGFR: Estimated glomerular filtration rate
HER: Electronic health record
ICU: Intensive care unit
MDRD: Modification of Diet in Renal Disease
MRSA: Methicillin-resistant Staphylococcus aureus
q8h: Every 8 h
VA-AKI: Vancomycin associated Acute Kidney Injury

## Footnotes

1 The publication regarding the performance of this model is under review.
