# Supplementary material for: Discrepancy between perceptions and acceptance of clinical decision support Systems: implementation of artificial intelligence for vancomycin dosing
Source: BMC Med Inform Decis Mak. 2023 Aug 11;23:157. doi: 10.1186/s12911-023-02254-9 (PMC10416522; doi:10.1186/s12911-023-02254-9)
Supplement: Supplementary file 1 — Additional file 1: Appendix 1. [file 12911_2023_2254_MOESM1_ESM.docx]

**Questionnaire for Pharmacist perceptions on the use of Artificial Intelligence to determine the dose of vancomycin**

| **Part 1.** Demographic characteristics |
| --- |
| Q1. Your age |
|  |
| Q2. Gender |
| 1. Female 2. Male |
| Q3. Are you Central Pharmacist or Critical Care Pharmacist? |
| 1. Central Pharmacist 2. Critical Care Pharmacist |
| Q4. How many years have you worked as Critical Care or Central Pharmacist? |
| A. 0-5 years  B. 6-10 years  C. 11-15 years  D. > 15 years |
| Q5. Assuming you have an average census for patients newly started on vancomycin during your shift, on how many occasions do you review or re-review their vancomycin regimens (across all of these new starts)? |
| A. 0-5 times  B. 6-10 times  C. 11-15 times  D. > 15 times |
| Q6. Assuming you have an average census for patients stabilized on vancomycin before your shift, on how many occasions do you review or re-review their vancomycin regimens (across all of these treated patients)? |
| A. 0-5 times  B. 6-10 times  C. 11-15 times  D. > 15 times |
| Q7. How long does it take you to determine a dose of vancomycin for a new patient? |
| A. 0-10 minutes  B. 11-20 minutes  C. 21-30 minutes  D. > 30 minutes |
| Q8. How long does it take you to review and, if appropriate, revise a regimen for the patients who are already on vancomycin therapy? |
| A. 0-5 minutes  B. 5-10 minutes  C. 11-15 minutes  D. > 15 minutes |
| Q9. What percentage of the time do you need to change the dose entered by the ordering provider? |
| A. 0-10%  B. 11-20%  C. 21-30%  D. > 30% |
| Q10. Which formula do you use to dose vancomycin? |
| A. CrCI, by Cockroft-Gault  B. eGFR_Cr_, by MDRD or CKD-EPI  C. eGFR_CysC_, by CKD-EPI  D. eGFR_Cr-CysC_, by CKD-EPI  E. Other ( ) |
| **Part 2.** Attitude to AI |
| Q11. To what extent do you agree that artificial intelligence (AI) can potentially effect your dosing decisions for vancomycin? |
| A. Strongly agree  B. Agree  C. Neither agree nor disagree  D. Disagree  E. Strongly disagree |
| Q12. To what extent do you agree that AI could enhance your ability to make vancomycin dosing decisions? |
| A. Strongly agree  B. Agree  C. Neither agree nor disagree  D. Disagree  E. Strongly disagree |
| Q13. To what extent do you agree that you would use AI when making dosing decisions? |
| A. Strongly agree  B. Agree  C. Neither agree nor disagree  D. Disagree  E. Strongly disagree |
| Q14. To what extent do you agree that your performance would be superior if using AI on a routine basis? |
| A. Strongly agree  B. Agree  C. Neither agree nor disagree  D. Disagree  E. Strongly disagree |
| **Part 3.** Share the cases |
| Q15. Please provide a dosing scheme for this case. |
|  |
| Q16. (Share what dose patients actually received)  Why do you think your prediction is similar or different from doses the patient actually received? |
|  |
| Q17. (Share prediction of under-or overdosing by AI)  Do you want to change your dosing scheme? |
| A.yes  B.no |
| Q18. (If yes) Can you talk about why do you want to change your dosing scheme?  (If no) Can you talk about why you do not want to change your dosing scheme? |
|  |
| Q19. (Share the dosage that AI provides)  Do you agree with the dose that AI provides? |
| A.yes  B.no |
| Q20. (If yes) Can you talk about why you agree with the dose that AI provides?  (If no) Can you talk about why you disagree with the dose that AI provides? |
|  |
| Q21. Can you give some advice about including the AI within the pharmacists’ workflow to enhance compliance? |
|  |
